# An efficient classification method based on principal component and sparse representation

**DOI:** 10.1186/s40064-016-2511-z

**Published:** 2016-06-22

**Authors:** Lin Zhai, Shujun Fu, Caiming Zhang, Yunxian Liu, Lu Wang, Guohua Liu, Mingqiang Yang

**Affiliations:** School of Mathematics, Shandong University, Shanda Nanlu 27, Jinan, 250100 China; School of Computer Science and Technology, Shandong University of Finance and Economics, Jinan, 250061 China; School of Computer Science and Technology, Shandong University, Jinan, 250101 China; School of Public Health, Shandong University, Jinan, 250012 China; Department of Ophthalmology, Qilu Children’s Hospital of Shandong University, Jinan, 250022 China; School of Information Science and Engineering, Shandong University, Jinan, 250100 China

**Keywords:** Palmprint recognition, Image classification, Principal component analysis, Sparse representation, Subspace optimization

## Abstract

As an important application in optical imaging, palmprint recognition is interfered by many unfavorable factors. An effective fusion of blockwise bi-directional two-dimensional principal component analysis and grouping sparse classification is presented. The dimension reduction and normalizing are implemented by the blockwise bi-directional two-dimensional principal component analysis for palmprint images to extract feature matrixes, which are assembled into an overcomplete dictionary in sparse classification. A subspace orthogonal matching pursuit algorithm is designed to solve the grouping sparse representation. Finally, the classification result is gained by comparing the residual between testing and reconstructed images. Experiments are carried out on a palmprint database, and the results show that this method has better robustness against position and illumination changes of palmprint images, and can get higher rate of palmprint recognition.

## Background

As an important application in optical imaging, palmprint recognition devices screen individual status by extracting effective textures of human palm (Shu and Zhang 1998; Feng et al. 2015; Fei et al. 2016). Compared with other optical recognition methods, such as fingerprint, face and gait, palmprint recognition has many advantages including low price of capture devices, low offensive and fixed rich texture features, which make it become a research focus in optical imaging and perception (Shu and Zhang 1998; Kong et al. 2009; Zhang et al. 2012).

Key issues in palmprint recognition are feature extraction and classification. The subspace method is one of main methods for feature extraction, including principal component analysis (PCA), independent component analysis (ICA), linear discriminant analysis (LDA), and so on (Connie et al. 2003; Duda et al. 2012; Zabalza et al. 2014; Ford et al. 2015). Among these methods, the most classic algorithm is the principal component analysis (Belhumeur et al. 1997), where original image matrix is converted into an one-dimensional vector, and limited features are used as accurately as possible to represent original image. However, its disadvantage is that the process of image matrix being converted into one-dimensional vector will cause problems like loss of spatial information. On the basis of this, the two-dimensional principal component analysis (2DPCA) method proposed by Yang et al. (2004) overcomes this defect well, which operates the image matrix directly instead of converting it in advance, but the dimension of feature vector is still high. Zhang and Zhou (2005) proposed the bi-directional two-dimensional principal component analysis ($\hbox {(2D)}^{2} \hbox {PCA}$) method, which extracts features from both row and column respectively, reducing the correlation between row and column, and also reducing the dimension of image feature matrix. As a result, the recognition rate is much improved (Pan and Ruan 2008).

The traditional signal sampling must follow the Nyquist sampling theorem: in order to reconstruct an analog signal without distortion, the sampling frequency should not be less than two times of the highest frequency of the signal spectrum (Gonzalez and Woods 2004). Compressed sensing (CS) theory was proposed by Donoho (2006), Candès and Wakin (2008), which has broken the restriction of the traditional Nyquist sampling theorem, and has brought a revolutionary change to the field of signal processing. In compressed sensing one can acquire the discrete samples of signal, where the sampling rate is far less than that of the Nyquist sampling, while still ensuring no distortion in the reconstructed signal under certain conditions. If observation matrix and sparse signal are known, sparse representation of original signal will be obtained. This sparse representation can be thought of as a compressed coding of the original signal, and the coded signal can be the basis for classification in the context of palmprint recognition (Wright et al. 2010). Sparse representation based classification (SRC) has been widely applied to the field of biological feature recognition (Wright et al. 2009; Yin et al. 2016; Feng et al. 2015). In this framework, however, the computational complexity of the reconstruction by the L1 norm minimization is very high, consequently a lot of time and space resources are needed in the process of numerical solution.

In view of the influence on palmprint recognition rate of unfavorable factors such as palm position, illumination, capture devices, etc, and the high computational complexity of traditional sparse classification methods, this paper presents fusion of blockwise bi-directional two-dimensional principal component analysis and grouping sparse representation-based classification method. In this method, a palmprint image is first divided into equal blocks, which can make image information more fully utilized; then, $\hbox {(2D)}^{2} \hbox {PCA}$ is used for each block to reduce dimension and to build an overcomplete dictionary; finally, a special subspace orthogonal matching pursuit algorithm is designed to solve the grouping sparse representation to obtain a final classification. The palmprint image is preprocessed before palmprint recognition, which can largely solve the above problem of the unfavorable factors. In addition, the method of the $\hbox {(2D)}^{2} \hbox {PCA}$ with image blocking used in the recognition stage, still can partly overcome the difficulties: $\hbox {(2D)}^{2} \hbox {PCA}$ can better extract image information from both rows and columns, which reflects more accurate features of image than PCA, 2DPCA and the random projection method; image blocking has good adaptability to the changes in posture and illumination (Gottumukkal and Asari 2004). It is explained as follows. Most dimensionality reduction based palmprint recognition measure the global information of each palmprint image and express them with a set of weights (feature), so they are not very effective in the case of changing position and illumination. Weight vectors will be greatly affected by the conditions from the weight vectors of the image with normal position and illumination, therefore it is hard to identify them accurately. If the palmprint image is divided into smaller blocks and the weight vector of each block is calculated, the local information of the palmprint can be well represented by the weights. Once the position or the illumination changes, just some of palmprint blocks will vary and the rest of the blocks will remain the same as the normal palmprint blocks, so the category can still be determined accurately by the remaining blocks.

We organize the remaining part of this paper as follows. In the “Principal component and sparse representation” section, principal component analyse with $\hbox {(2D)}^{2} \hbox {PCA}$, sparse representation for compressed sensing and sparse classification are reviewed respectively. In the “Our method” section, feature extraction by blockwise bi-directional two-dimensional principal component analysis and our grouping sparse classification are described in detail. In the “Experimental results and analysis” section, experiments on data of a special palmprint database are implemented to verify advantages of our proposed algorithm. We give the conclusions of this paper in the “Conclusions” section.

## Principal component and sparse representation

### $\hbox {(2D)}^{2} \hbox {PCA}$

In the $\hbox {(2D)}^{2} \hbox {PCA}$ method feature matrix is extracted from two directions of both row and column of image respectively, which can reduce the correlation between them and the dimension of the feature matrix of image.

Assuming *N* is the number of image sample category, there are $n_i$ images with size $l\times h$ in the *i*th category, and the total number of samples is $n=\sum _{i=1}^N n_i$. For a learned projection matrix $X (h\times t)$ obtained from row direction, and a learned projection matrix $Z (l\times s)$ obtained from column direction from a set of training images, we project the original image $A (l\times h)$ onto *X* and *Z* in sequence, thus generating a $s\times t$ dimensional coefficient (feature) matrix $C=Z^TAX$. Conversely, a reconstructed image $\hat{A}$ can be obtained by using the coefficient matrix for image reconstruction (Zhang and Zhou 2005):

1 $$\begin{aligned} \hat{A}=ZCX^T. \end{aligned}$$

### Sparse representation for compressed sensing

The compressed sensing theory tells us that, if a signal is sparse or compressible through an orthogonal transformation, the signal can be observed in a lower frequency, and can be represented with least numbers of observation values. Moreover, the original signal can be estimated well by these sparse observation values (Donoho 2006; Candès and Wakin 2008).

In the case of one dimension, suppose *x* is an original signal with length *n*, *y* is an observed signal with length *m*, and $D (m\times n, m\ll n)$ is a measurement matrix satisfying

2 $$\begin{aligned} y=Dx. \end{aligned}$$

Under certain conditions *x* can be sparsely reconstructed from *y* through solving the following L0 norm optimal problem (Elad 2010):

3 $$\begin{aligned} \hat{x}=argmin\Vert x\Vert _0, \quad s.t.\quad Dx=y. \end{aligned}$$

There are a large number of elaborated work to solve fast above problem, for example the orthogonal matching pursuit (OMP) algorithm (Tropp and Gilbert 2007; Elad 2010).

### Sparse classification

A supervised classification is to use labeled training samples from special object categories to correctly determine the category to which a new test sample belongs. The basic idea of sparse representation-based classification (SRC) method is as follows. Assuming that a test sample can be linearly represented by training samples obtained from its same category, one assembles an overcomplete dictionary composed of all training samples from object categories, and lets a test sample project on it. Because the test sample only has bigger coefficients corresponding to some sample category in the aforementioned dictionary representation, the test sample is usually sparse in the representation of the overcomplete dictionary. According to this sparse representation the test sample can be correctly classified.

Given *N* categories of gray palmprint image with size $l\times h$, and there are $n_i$ training samples in the *i*th category. If each image is converted into a column vector $\nu \in R^m (m=l\times h)$, the image *y* to be identified in the *i*th category can be expressed as (Wright et al. 2009):

4 $$\begin{aligned} y=\alpha _{i,1}\nu _{i,1}+\alpha _{i,2}\nu _{i,2}+\cdots +\alpha _{i,n_i}\nu _{i,n_i}, \end{aligned}$$

where $\alpha _{i,j}\in R$ and $\nu _{i,j}\in R^m, j=1,2,\ldots n_i$, are scale factors and column vectors belonging to the *i*th category, respectively.

We construct a training sample matrix of *N* categories $D=[D_1,D_2,\ldots ,D_N]\in R^{m\times n}$ with the sample dimension *m* and the number of total samples $n (= \sum _{i=1}^N n_i)$, where $D_i=[\nu _{i,1},\nu _{i,2},\ldots ,\nu _{i,n_i}]$ is a group of the training samples in the *i*th category. Any sample *y* to be identified can be linearly represented through *D*:

5 $$\begin{aligned} y=Dx. \end{aligned}$$

Thus, the pattern recognition problem is converted into to solve the equation (5) (Wright et al. 2009).

Ideally, for an image *y* belonging to the *i*th category, there should be a corresponding vector $x=[0,0,\ldots 0,\alpha _{i,1}, \alpha _{i,2},\ldots ,\alpha _{i,n_i},0,0,\ldots 0,]^T\in R^n$ according to the Eq. (5). Moreover, if the total number of samples is greatly larger than the number of samples in each category: $n\gg max(n_i)$, the proportion of nonzero elements in $x \, n_i/n$ will be much smaller. The greater the difference between *n* and $max(n_i)$, the sparser *x* is, and the more favorable it is to the sparsity classification.

But, in actual process of palmprint recognition, the testing samples may be subjected to changes of position and illumination and other factors. Consequently, nonzero elements may appear in other locations, and the Eq. (5) reduces into:

6 $$\begin{aligned} y=Dx+e. \end{aligned}$$

Here, *e* is an error vector representing changes of position and illumination of testing samples.

From the above discussion, however, if the overcomplete dictionary *D* consists of all training samples, it can be expected that any testing sample is sparse in *D* in any case, and is only similar to the elements of *D* belonging to the same category.

## Our method

### Blockwise bi-directional two-dimensional principal component analysis

The dimension of a palmprint image is usually very high after being converted into one-dimensional vector. In order to solve *y* in the Eq. (5), one needs to solve a high-dimensional linear equations, which is very difficult in actual applications. In this paper, we propose a blockwise bi-directional two-dimensional principal component analysis to reduce the dimension of image. To be specific, image blocking and the $\hbox {(2D)}^{2} \hbox {PCA}$ method are combined to extract palmprint image features, which divides the image to be identified into some subimages, and then identifies the subimages with $\hbox {(2D)}^{2} \hbox {PCA}$. Because changes of position and illumination only influence a few subimages, and do not influence all subimages, this method can effectively overcome negative effects of position and illumination changing in traditional PCA algorithms. Finally, the reduced and normalized feature matrixes are converted into column vectors to assemble an overcomplete dictionary for the sparsity classification.

In order to get optimal projection vectors, the original image matrix $A (l\times h)$ is divided into $p\times q$ blocks:

7 $$\begin{aligned} A=\left( \begin{array}{cccc} A_{11} & \quad A_{12} & \quad \ldots &\quad A_{1q}\\ A_{21} & \quad A_{22} & \quad \ldots &\quad A_{2q}\\ \vdots & \quad \vdots & \quad \ddots &\quad \vdots \\ A_{p1} & \quad A_{p2} & \quad \ldots &\quad A_{pq}\\ \end{array} \right) . \end{aligned}$$

Here, the size of each subimage is $l_1\times h_1 (p\times l_1=l, q\times h_1=h)$, on which the $\hbox {(2D)}^{2} \hbox {PCA}$ transform is used, and a feature matrix characterizing palmprint features is obtained as follows:

8 $$\begin{aligned} B=\left( \begin{array}{cccc} Z_{11}^TA_{11}X_{11} & \quad Z_{12}^TA_{12}X_{12} & \quad \ldots & \quad Z_{1q}^TA_{1q}X_{1q}\\ Z_{21}^TA_{21}X_{21} & \quad Z_{22}^TA_{22}X_{22} & \quad \ldots & \quad Z_{2q}^TA_{2q}X_{2q}\\ \vdots & \quad \vdots & \quad \ddots & \quad \vdots \\ Z_{p1}^TA_{p1}X_{p1} & \quad Z_{p2}^TA_{p2}X_{p2} & \quad \ldots & \quad Z_{pq}^TA_{pq}X_{pq}\\ \end{array} \right) . \end{aligned}$$

**Fig. 1:**
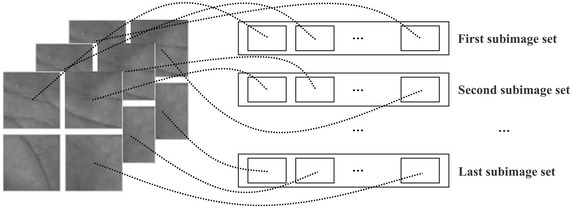
Formation of subimage sets for blockwise PCA with blocking number $2\times 2$

Then, subimage sets can be built, which are composed of subimages in same positions. An example with the blocking number $2\times 2$ is shown in Fig. 1. In particular, when the blocking number is $1\times 1$, above blockwise bi-directional two-dimensional principal component analysis ($\hbox {B(2D)}^{2} \hbox {PCA}$) degenerates into the original $\hbox {(2D)}^{2} \hbox {PCA}$.

The proposed method can effectively reduce the computational complexity. For each subimage block from a palmprint image, its size is $l_1\times h_1$. If one reserves $p (p<\{l_1, h_1\})$ eigenvalues in the PCA transformation, then the size of a subimage with dimensionality reduction by $\hbox {(2D)}^{2} \hbox {PCA}$, is $p\times p$; the size of a subimage by 2DPCA is $l_1\times p$; although the size by PCA is $p\times 1$, one need to convert the image into one-dimensional matrix before image projection, which means the size of each subimage is $l_1h_1\times 1$. As one can see, the $\hbox {(2D)}^{2} \hbox {PCA}$ consums the least computer memory. Thus, $\hbox {(2D)}^{2} \hbox {PCA}$ used for dimensionality reduction can effectively reduce the computational complexity compared with PCA and 2DPCA.

### Grouping sparse classification

In order to speed up the process of sparsely solving of the Eq. (5), we employ a subset technique [(subspace orthogonal matching pursuit (SOMP)] to solve it in each training category, which will effectively reduce the computational cost by canceling a great number of columns of *D*.

Specifically, for the total category number *N* of training samples, our arithmetic steps are as follows:

1. The training sample matrix is divided into blocks of equal size to form subimage sets, which are composed of subimages in the same positions after being divided; dimension reduction and normalization with $\hbox {(2D)}^{2} \hbox {PCA}$ are used for each subimage; an overcomplete dictionary $D'$ is formed, and according to the sample category, $D'$ is divided into *N* training submatrixes:
  9 $$\begin{aligned} D'_1,D'_2,\ldots D'_N. \end{aligned}$$
  That is $$D'=[D'_1,D'_2,\ldots ,D'_N]$$.
2. For each test sample $y, y=D'_ix_i,i=1,2,\ldots , N$, is solved by the orthogonal matching pursuit algorithm, and the coefficient vector $[x_1,x_2,\ldots ,x_N]$ is obtained.
3. The recognition coefficient $x_i$ of each category is related to the testing sample to calculate the residual:
  10 $$\begin{aligned} r_i=\Vert y-D'_ix_i\Vert _2,\quad i=1,2,\ldots ,N. \end{aligned}$$
4. Finally, a recognition result is outputted:
  11 $$\begin{aligned} Identity(y)=argmin(r_i),\quad i=1,2,\ldots ,N, \end{aligned}$$
  where, the category of the minimum reconstruction error is just that of the test data *y*.

The method SOMP is used to greatly reduce the size of the calculated sample, which makes it easy to calculate sparse coefficients and error results in the comparison, and reduces the number of the loop count accordingly, improving the precision level than the calculation of samples with larger size. More important, this method can significantly improve the recognition result.

## Experimental results and analysis

In order to verify advantages of the proposed method using the dimension reduction with $\hbox {B(2D)}^{2} \hbox {PCA}$ and the subspace orthogonal matching pursuit (SOMP), three experiments are carried out according to different dimension reductions, sparse classifications and image blockings.

**Fig. 2:**
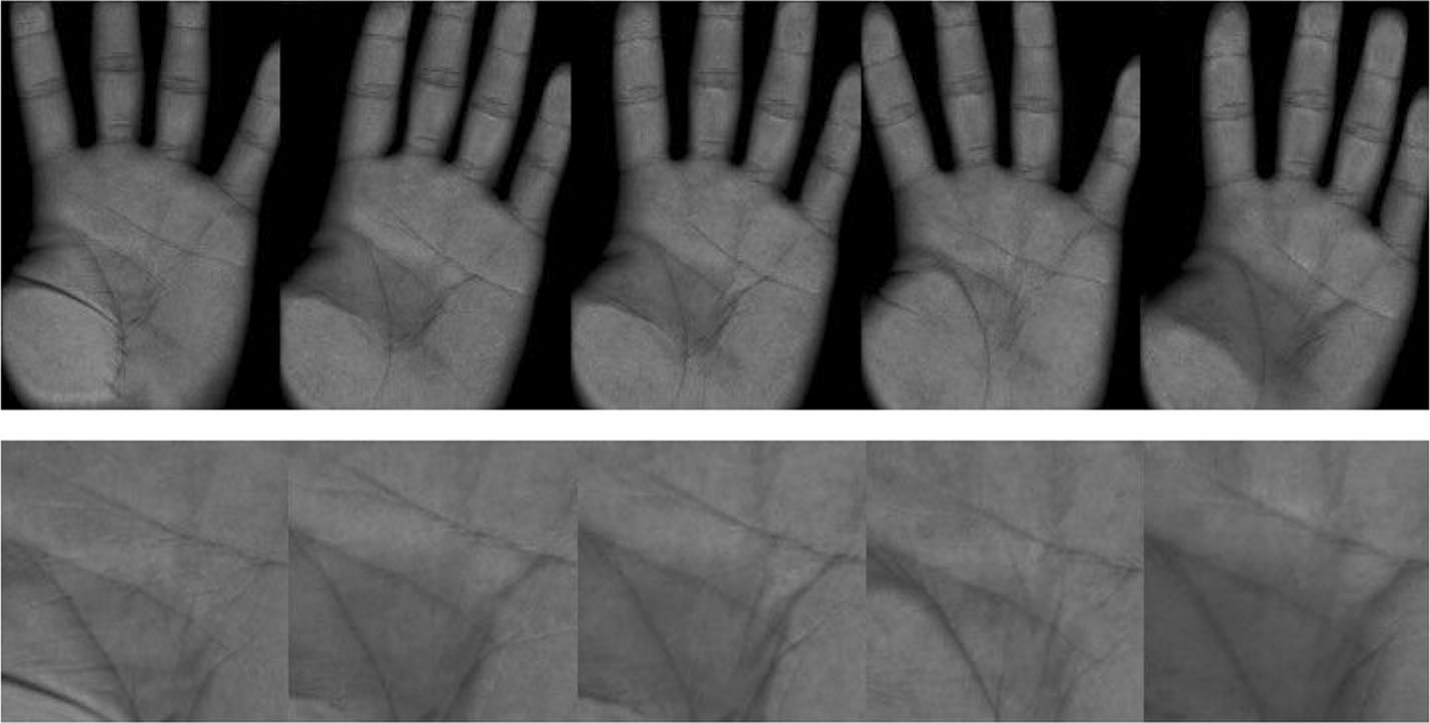
Part of palmprint data from Beijing Jiaotong University: original images (*top*) and their regions of interest (*bottom*)

All images in following experiments are from a palmprint database collected by an optical plamprint scanner in Beijing Jiaotong University (Pan and Ruan 2009), including 500 gray palmprint images of 50 individuals, where each individual has 10 images. The resolution of original image is $413\times 292$, with different changes of location and illumination. Part of original palmprint images and their regions of interest (ROI) are shown in Fig. 2. The images in the palmprint database are divided into training and testing samples. For each person, first five images are as training samples, last five images are as testing samples. In order to compare the recognition rate of each algorithm, all methods are implemented using the MATLAB programming under current Windows operating systems.

### Experiment 1: recognition rates with different dimension reduction methods

Different dimension reduction methods are compared. Random projection: test image is converted into a column vector, and is projected onto a random Gaussian matrix, and then feature vectors are obtained. PCA: test image is also converted into a column vector and is reduced in the dimension. $\hbox {(2D)}^{2} \hbox {PCA}$: the dimension of test image is reduced by the bi-directional projection, and then the feature matrix is obtained. $\hbox {B(2D)}^{2} \hbox {PCA}$: test image is first divided into 16 subimages with the blocking number $4\times 4$, and then $\hbox {(2D)}^{2} \hbox {PCA}$ is used to reduce the dimension of each subimage to get the feature matrix. In the final step, the subspace orthogonal matching pursuit (SOMP) is used for all of above feature data to complete corresponding sparse classifications.

**Fig. 3:**
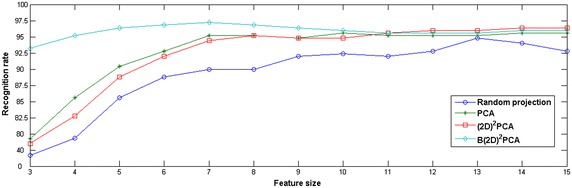
Recognition rates ($\%$) using SOMP classification with different dimension reduction methods

In Fig. 3, recognition rates are shown in sparse classifications with different dimension reduction methods. Here, the size of one side of square feature matrix is defined as feature size, which means feature dimension is the double of the feature size. It can be seen that, when the feature size is lower than 12, the recognition rate using $\hbox {B(2D)}^{2} \hbox {PCA}$ is always higher than that of anyone of other three methods. When the feature size is 7, $\hbox {B(2D)}^{2} \hbox {PCA}$ has reached the optimal recognition rate of 97.2 %; at this time, $\hbox {(2D)}^{2} \hbox {PCA}$ is 94.4 %, PCA is 95.2 %, and the random projection is only 90 %. After the feature size is 12, recognition rates of both $\hbox {(2D)}^{2} \hbox {PCA}$ and $\hbox {B(2D)}^{2} \hbox {PCA}$ tend to be consistent having reached above 96 %. When the feature size is 14, $\hbox {(2D)}^{2} \hbox {PCA}$ achieves the optimal recognition rate of 96.4 %.

In Table 1, four dimension reduction methods are compared in respect of optimal recognition rate and corresponding feature dimension. It is clear that, the proposed method achieves the highest recognition rate of 97.2 % with the smallest feature dimension of 49 among four methods, guaranteeing better robustness against changes of palmprint position and illumination.

**Table 1:** Optimal recognition rates (%) and corresponding feature dimensions with different dimension reduction methods

Dimension reduction method	Feature dimension	Optimal recognition rate
Random projection	225	94.8
PCA	100	95.6
$\hbox {(2D)}^{2} \hbox {PCA}$	196	96.4
$\hbox {B(2D)}^{2} \hbox {PCA}$	49	97.2

### Experiment 2: recognition rates with different classification methods

The $\hbox {B(2D)}^{2} \hbox {PCA}$ algorithm is first used to reduce the dimension of test image to get the feature vector, followed by a normalizing processing; then, both OMP and SOMP methods are used for sparse classification and recognition.

**Fig. 4:**
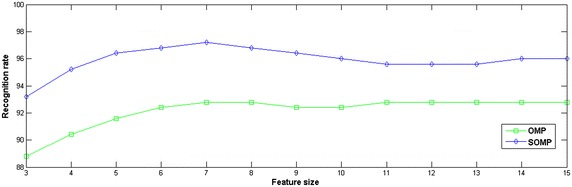
Recognition rates (%) using different classification methods

In Fig. 4, recognition rates are shown in sparse classifications using different methods of solving the Eq. (5). It can be seen that, SOMP has more advantages than OMP: its corresponding recognition rate is always higher than that of the latter at least two percentage points. This is because, training samples are divided into subblocks firstly, and then the OMP algorithm is used on them, which reduces the data dictionary in scale obtained from training subsamples, so that the solution of the Eq. (5) is much closer to the ideal linear coefficients, which further improves the calculating precision. Therefore, the SOMP algorithm can effectively improve the classification performance of palmprint recognition.

### Experiment 3: recognition rates with different blocking numbers

Here, we discuss the effect of feature dimension on the recognition, where different blocking numbers: $1\times 1$ (without dividing), $2\times 2, 2\times 4, 4\times 4$ and $8\times 8$ are compared. SOMP is used in the sparse classification.

**Fig. 5:**
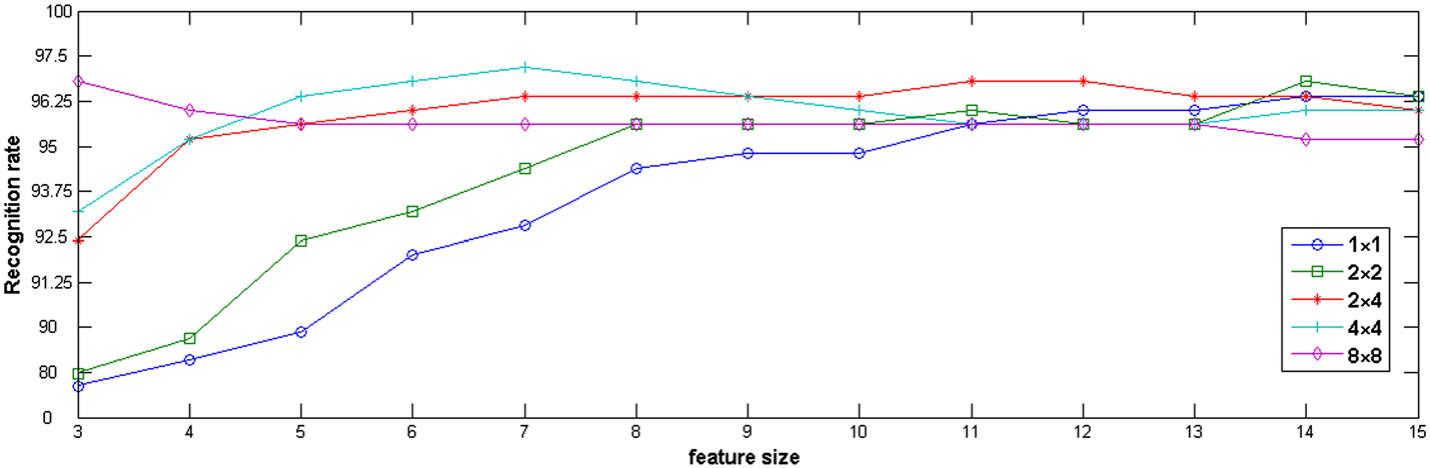
Recognition rates (%) using SOMP classification with different blocking numbers

In Fig. 5, recognition rates in different blocking numbers are shown. It can be seen that, when the feature dimension is low, the recognition rate with the dividing technique is higher than that of non-dividing in same conditions; when the feature size is 7, the sparse classification with $4\times 4$ blocking reaches the highest recognition rate of 97.2 %, which shows that the combination of blocking method and $\hbox {(2D)}^{2} \hbox {PCA}$ is much effective. The reason for this advantage can be explained as follows: the subimage is smaller in size after image dividing, and one can extract more feature details which is conducive to palmprint recognition; at the same time, when the palmprint image is disturbed by position, illumination and other external factors, only a small portion of subimages will be influenced, other subimages will not be done.

With the increase of feature size, the recognition rates of most blocking numbers gradually increase. When the blocking number reaches $4\times 4$ and the feature size is 7, the recognition rate reaches the peak for all blocking numbers. When the blocking number increases to $8\times 8$, its recognition rate is higher than that of $4\times 4$ at the first two feature dimensions, but it subsequently shows a downward trend and it can not exceed the case of $4\times 4$ any more. It can be explained that when the number of blocks is too small (less than $4\times 4$), the advantage of robust against changed position and illumination can not be highlighted completely; when the number of blocks is too big (more than $4\times 4$), this may get good result at low feature dimensions. However, overmuch blocks will undermine the overall structure information of palmprint images, resulting in the drop of recognition rate, and the recognition time will also increase significantly. Of course, for different palmprint database, due to the different image size and block mode, one need concrete analysis on a case-by-case basis.

In Fig. 5, it also can be seen that, when the blocking number is too small, each subimage will be bigger in size, as a result this method can not display the strength of being insensitive to position and illumination changes; when the blocking number is too big, on the contrary, each subimage will be much smaller, which will damage the global structure information of the palmprint image, resulting in the decreasing of recognition rate. Therefore, one should select appropriate blocking number and feature dimension based on different degradations of palmprint image when utilizing $\hbox {B(2D)}^{2} \hbox {PCA}$ to extract feature vectors.

## Conclusions

In this paper an efficient grouping sparse classification is proposed with dimension reduction using robust blockwise principal components as feature vectors, which greatly reduces the feature dimension and overcomes interferences from unfavorable external factors, obtaining better recognition results in the process of palmprint recognition. Obvious advantages in both recognition rate and reduction of feature dimension are verified in experiments on special palmprint data in the comparison of some related methods. In the case of noise corruption palmprint enhancement for more effective dictionary using fringe filtering (Fu and Zhang 2012) is one of ongoing work in grouping sparse classification for degraded palmprint images.
